# Microglia and extracellular vesicle‐mediated spread of Tau pathology

**DOI:** 10.1002/alz.087779

**Published:** 2025-01-03

**Authors:** Tsuneya Ikezu

**Affiliations:** ^1^ Mayo Clinic, Jacksonville, FL USA

## Abstract

**Background:**

Extracellular vesicles (EVs) carry pathogenic molecules and play a role in the disease spread, including aggregated tau proteins. The Endosomal Sorting Complexes Required for Transport (ESCRT) machinery is responsible for the biogenesis of small EVs (exosomes), thus targeting critical ESCRT molecules can disrupt EV synthesis. We hypothesize that microglia‐specific targeting of ESCRT‐I molecule Tsg101 suppresses microglia‐derived EV‐mediated propagation of tau pathology, leading to amelioration of the disease phenotype of the tauopathy mouse model.

**Methods:**

PS19 tau transgenic mouse line was crossed with Cx3cr1^CreERT2/+^:Tsg101^fl/fl^ lines for tamoxifen‐inducible Cx3cr1‐specific deletion of *Tsg101* (Tsg101 cKO) in PS19 mice to generate WT, Tsg101 cKO, PS19, and PS19:Tsg101cKO groups. The animals were treated with tamoxifen or corn oil at 2 months of age and subjected to comprehensive behavioral, neuropathological, biochemical, and molecular biological assessments at 6‐7 months of age. The microglia isolated from Cx3cr1^CreERT2/+^:Tsg101^fl/fl^ pups were subjected to in vitro synaptosome uptake analysis with or without 4‐Hydroxytamoxifen treatment.

**Results:**

PS19 mice develop cognitive impairment as determined by Y‐maze, forced alternation, novel object recognition and fear conditioning, which are reversed in PS19:Tsg101cKO mice. This is correlated with reduced Alz50^+^ tau accumulation, neurodegenerative microglial activation, neuroinflammation and complement pathway activation as determined by bulk RNA sequencing, ELISA and neuropathology. Primary cultured Cx3cr1^CreERT2/+^:Tsg101^fl/fl^ microglia with 4‐Hydroxytamoxifen treatment show reduced phagocytosis of *E. coli* particles and synaptosome in C1q dependent manner. Tsg101 cKO microglia show reduced expression of C3aR1 and CD68, and secretion of total and Tau^+^ EVs in vivo.

**Conclusion:**

Microglia‐specific targeting of Tsg101 show beneficial effect for ameliorating the disease progression of tauopathy mouse model via suppression of EV secretion, microglial activation, tau accumulation and complement‐dependent synaptic pruning. Microglial Tsg101 is a potential therapeutic target of Alzheimer’s disease and related tauopathy.

